# Denoising of BOTDR Dynamic Strain Measurement Using Convolutional Neural Networks

**DOI:** 10.3390/s23041764

**Published:** 2023-02-04

**Authors:** Bo Li, Ningjun Jiang, Xiaole Han

**Affiliations:** 1 Institute of Geotechnical Engineering, Southeast University, Nanjing 211189, China; 2 Department of Engineering, University of Cambridge, Cambridge CP2 1PZ, UK; 3 Department of Civil and Environmental Engineering, University of Hawaii at Manoa, Honolulu, HI 96822, USA

**Keywords:** Brillouin scattering, fibre optic sensing, image denoising, convolutional neural network

## Abstract

The Brillouin optical time domain reflectometry (BOTDR) system measures the distributed strain and temperature information along the optic fibre by detecting the Brillouin gain spectra (BGS) and finding the Brillouin frequency shift profiles. By introducing small gain stimulated Brillouin scattering (SBS), dynamic measurement using BOTDR can be realized, but the performance is limited due to the noise of the detected information. An image denoising method using the convolutional neural network (CNN) is applied to the derived Brillouin gain spectrum images to enhance the performance of the Brillouin frequency shift detection and the strain vibration measurement of the BOTDR system. By reducing the noise of the BGS images along the length of the fibre under test with different network depths and epoch numbers, smaller frequency uncertainties are obtained, and the sine-fitting R-squared values of the detected strain vibration profiles are also higher. The Brillouin frequency uncertainty is improved by 24% and the sine-fitting R-squared value of the obtained strain vibration profile is enhanced to 0.739, with eight layers of total depth and 200 epochs.

## 1. Introduction

Due to the wide range of applications in structural health monitoring and the advantages of stability and flexibility in harsh environments, the Brillouin scattering-based distributed fibre optic sensors have attracted significant attention in recent decades. These sensors can quantitatively measure the distributed strain and temperature information along the optic fibre. The Brillouin optical time domain reflectometry (BOTDR) has the advantages of single-end access and simple operation at the construction site, which makes it very popular in civil engineering applications. Furthermore, the demand for the dynamic sensing of strain information is becoming stronger and stronger, along with the rapid developments in some civil engineering fields such as geophysical sciences and the oil and gas industries. Therefore, the dynamic measurement of strain using the BOTDR system is a practical and promising topic, especially for civil engineering-related industries.

The BOTDR system obtains the distributed temperature and strain information along the optic fibre under test (FUT) by sensing distributed Brillouin frequency shift (BFS) profiles. The BFS profiles are extracted by finding the peak values of each Brillouin gain spectrum (BGS) along the FUT. Therefore, the signal-to-noise ratio (SNR) of the BGSs limits the performance of the BOTDR system. By introducing noise reduction, the system performance could be improved.

By treating the BGSs along the FUT as a two-dimensional image, noise suppression can be conducted on Brillouin-based distributed fibre optic sensors using image denoising methods [1,2,3]. It has been proved that image denoising methods are more effective compared with one-dimensional denoising methods such as one-dimensional wavelet denoising [4]. Non-local means (NLM) and wavelet denoising (WD) are the main image denoising methods used for Brillouin-based fibre optic sensors, as well as block-matching and 3D filtering (BM3D) [1,2,3,5,6,7,8]. However, these analytical image denoising methods suffer from degradation of frequency accuracy and spatial resolution, as details of the data are weakened and smaller data sampling point number leads to insufficient information for denoising [1,9]. The accuracy can be degraded by as much as about 14 MHz (1 °C approximate to 1 MHz) with NLM and about 11 MHz with WD [1]. In addition, NLM is highly dependent on the level of neighbouring data similarity. Furthermore, the performance of both NLM and BM3D relies on manual setup and parameter selection [1,8]. To overcome the drawbacks of analytical methods, image denoising methods based on deep learning have been proposed [10,11,12,13].

Deep learning on image denoising is popular and promising nowadays [14]. Deep learning finds the unknown solutions to tasks by learning from large datasets and does not require analytical definitions and prior knowledge of the tasks [15]. An artificial neural network (ANN) is a representative deep learning model [16], which is made up of a massive number of artificial neurons (computational elements) and weights that can be tuned. The architecture of a neural network typically consists of an input layer, hidden layers and an output layer. The input layer receives the input data, the hidden layers process the input (neurons as classifiers or feature detectors), and the output layer generates the results. The word “neural” is used here because the network was inspired by biological neurons. With more than one hidden layer, the ANN is described as a deep network, otherwise, a shallow network with only one hidden layer. The weights (and the biases) of an ANN are decided in the training process to produce the desired outputs. To tune the weights (and the biases) of an ANN, the neural network is trained over a large set of data, and the loss function, i.e., the difference between the predicted outputs and the actual outputs, is continuously computed. The weights and the biases are changed slightly until the lowest possible loss is achieved, which is known as backpropagation in deep learning [17].

The convolutional neural network (CNN) is at the heart of deep learning, which is capable of capturing the spatial dependencies in an image compared to an ANN. A CNN consists of an input layer, convolutional layers, pooling layers, one or more fully connected layers and an output layer. After being received by the input layer, the image is then convolved with a kernel (or called a filter) on the convolutional layer. The kernel moves across the image and is multiplied with the input image pixel by pixel over the part it is hovering. The convolved result is then summed with a bias and produces the feature output. With the kernel sliding over the image, the inputs share the same weights compared to ANN where each element of the weight is multiplied with only one element of the input and is never used again. This feature of parameter sharing also makes the model more efficient in terms of memory requirements and statistical efficiency. The pooling layer further reduces the spatial size of the feature output from the convolutional layer by returning one value from a specific part of the output from the last layer [18]. It makes the output unchanged even if the input is changed slightly. After the input data are converted to a desired form with the previous layers, the processed data are fed into a feedforward neural network and processed with a full connection between the input and output on the fully connected layer. The features of the image are then classified by a specified classification technique.

The image denoising methods with CNN are under rapid development [19,20] since AlexNet was proposed in 2012 [21]. The denoising method of CNN with residual learning (DnCNN) has realized significant improvement in the performance of image denoising [22]. Instead of producing the clean target image directly, DnCNN learns the noise and predicts the clean image by removing the noise from the noisy input. Due to the fact that the noise features are much different from the features of the noisy input, it is easier to learn the noise features than the clean image features and this leads to better noise removal. Compared with the image denoising methods, such as NLM and WD, DnCNN does not change the redundancy of the information. The data sampling point number is kept the same as it is of the input. Additionally, the frequency accuracy and spatial resolution are not degraded with DnCNN.

In this work, the denoising performance of DnCNN on the dynamic strain sensing of small gain stimulated Brillouin scattering (SBS) short-time Fourier transform (STFT)-BOTDR is experimentally demonstrated. Compared with conventional frequency scanning, STFT is faster and adds a noise removal effect. Small gain SBS boosts the Brillouin signal power of BOTDR and improves the SNR and system performance. The DnCNN performance on the detection of BGSs and distributed BFS profiles are experimentally validated. Experimental results of denoised BGS images, distributed BFS profiles, frequency uncertainties and the detection of strain vibration are compared, with different total depths and epochs of the training networks. 

## 2. Materials and Methods

Since CNN has superior performance in image recognition, the idea of image denoising based on CNN was later proposed by [22], named DnCNN. The aim of image denoising is to recover a clean image ***x*** from a noisy image ***y*** with noise ***v***. The relationship between them is ***y*** = ***x*** + ***v***, where ***v*** is commonly assumed to be additive white Gaussian noise (AWGN) with standard deviation σ. Instead of producing the clean image as the output, DnCNN predicts the noise as the residual image by residual learning and removes the latent clean image within the hidden layers. Since there is a larger difference between the input image and the residual noise, it is easier to learn the residual noise compared with the clean image.

### 2.1. DnCNN Architecture

**Figure 1:**
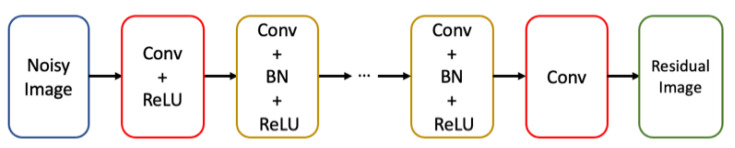
Architecture of DnCNN.

Figure 1 is the architecture of the DnCNN for noise learning. The input is the noisy image (***y*** = ***x*** + ***v***) to be processed. There are three types of hidden layers in this model. The first layer is the convolutional layer with rectified linear units (ReLU) for nonlinearity, which is to gradually separate the image structure from the noisy input. The second type is the convolutional layer with batch normalization (BN) and ReLU. The third type is the convolutional layer to reconstruct the output.

Batch normalization normalizes the inputs of each hidden layer by normalizing the means and the variances [18]. Mini-batch stochastic gradient descent (SGD) is a very popular tool in the training process of deep learning nowadays. The mini-batch method randomly chooses a subset of the training dataset to perform the backpropagation and evaluate its gradient. Compared with computing over the whole batch (batch gradient descent) or a single training sample (stochastic gradient descent SGD), mini-batch is a faster and more stable way to update the weights. During the training process with mini-batch, the distribution of a layer’s inputs changes due to the random initialisation of weights and change of the parameters as well as the randomness of the input data [23]. When updating the parameters by calculating the gradients, the layer has to continuously adapt to the new distribution. In experimental operations, the change in the means and variances of the inputs to layers during training processes is observed. This phenomenon is named internal covariate shift. Batch normalization is a method to mitigate the internal covariate shift. Arbitrary shift of data is removed by normalizing mean, while the arbitrary spread of the data is eliminated by variance normalization. With only two parameters added for each activation, batch normalization offers faster training and better convergence.

The ReLU is a widely used activation function to add non-linearity [24]. It trains fast, is expressive and prevents the gradient vanishing problem. The ReLU is expressed as

g(a) = max(a,0)(1)

**Figure 2:**
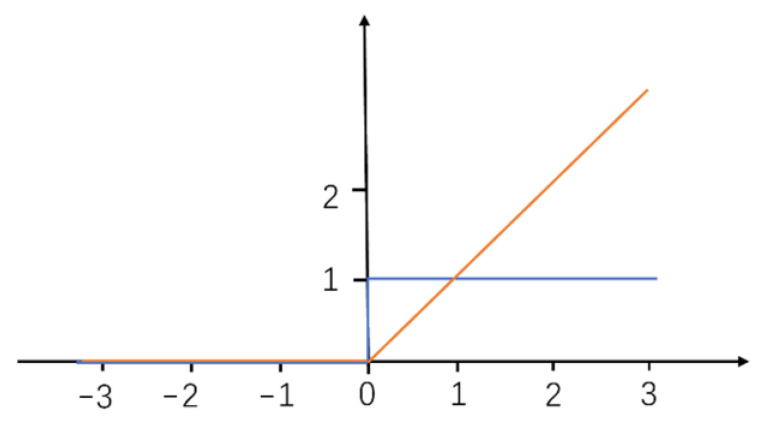
Rectified linear unit (ReLU) (red) and its derivative (blue).

Figure 2 is the ReLU and its derivative. It shows that, for input values below 0, ReLU returns the value 0, which adds nonlinearity and is very effective and filters out unwanted values (no small values left compared to some other activation functions). On the other hand, with input values greater than 0, ReLU works as a linear function. Its derivative is constantly 1, which is stable and eliminates the gradient vanishing problem. For the activation functions of derivative smaller than 1, the errors between the predicted values and the real values will be decayed as they propagate through the layers. As the network goes deeper, it will be harder for the model to converge, and the gradient vanishing problem becomes obvious.

Instead of training the mapping function of the clean image F(***y***) = ***x***, the target of the model is to train a residual mapping of noise R(***y***) = ***v***. The clean image can then be predicted by ***x*** = ***y*** − R(***y***).

The loss function of the model is written as

(2) $$l(\Theta )=\frac{1}{2N}\overset{N}{\underset{i=1}{\sum }}\|R\left(y_{i};\Theta \right)-{\left(y_{i}-x_{i})\right\|}_{F}^{2}$$

where *N* is the number of noisy–clean training image pairs [22].

This loss function learns the trainable parameters Θ by computing the averaged mean squared error between the estimated residual noise from the noisy input and the desired residual noise. In the training process, the DnCNN model extracts the features of the noise in the forward direction and minimizes the loss function by tuning the trainable parameters Θ in backpropagation.

Since the size of the input data shrinks as the data are convolved with the kernels on each hidden layer, zero-padding is added to keep the output size the same as the input size. Given the input size *i* (along both axes), the kernel size along both axes *k*, and zero padding parameter along both axes *p*, the output size *o* of a convolutional layer is calculated as [25]

*o* = (*i* − *k*) + 2*p* + 1 (3)

Having *o* = *i*, 2*p* = *k* − 1. For k to be odd,

*p* = (*k* − 1)/2 (4)

As the kernel size of 3 by 3 (*k* = 3) has been proven to be the most effective and widely used kernel size in CNN [20,26], 3 by 3 kernels are used here for image denoising. According to Equation (4), zero padding with *p* = 1 is added for *k* = 3 (1 row and column of 0 added on each boundary).

### 2.2. BOTDR Setup

**Figure 3:**
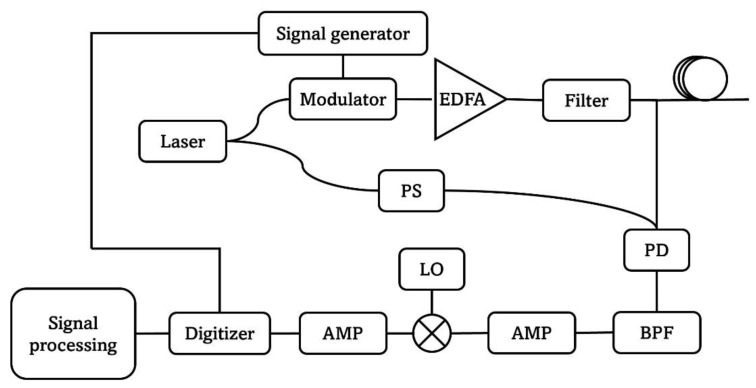
The BOTDR diagram.

To validate its performance, DnCNN is applied to the data processing of the small gain SBS STFT-BOTDR dynamic strain measurement as is shown in [27]. Figure 3 illustrates the BOTDR experimental setup. A narrow linewidth laser transmits continuous-wave (CW) light. The incident light is then equally split into two branches. In the upper branch, the light is modulated into a 40 ns pulse with 16 µs period. The modulator is controlled by a signal generator. The light pulse is amplified by an erbium-doped fibre amplifier (EDFA) and then goes through an optical bandpass filter to boost the signal power and reduce the noise after the amplification. The data with the input peak pulse power of 3.12 W are used in this work, as the optimal small gain SBS is induced at this input level. The incident light is sent into the FUT through a circulator and the backscattered light is delivered to the photodetector (PD). In the lower branch in Figure 3, a polarization scrambler (PS) is used to remove the effect of polarization mismatch between the two branches. The lower branch also goes to the PD so that the optical Brillouin signal is converted to the electrical signal on the PD. The signal is filtered by an electrical bandpass filter (BPF) and amplified by an amplifier (AMP). Since the Brillouin signal is around 11 GHz, which is a very high frequency, the electrical signal beats with an electrical local oscillator (LO) to down-convert the signal. After a second electrical amplifier (AMP), the signal is captured by a digitizer. The captured signal is processed by the signal processing method to reconstruct the BGS and BFS information. Each measurement is derived after 25 averaging, leading to 2.5 kHz sampling speed of the dynamic detection. In addition, the FUT is about 935 m long with the 60 Hz strain vibration added close to the far end of the fibre over about 6 m length. The fibre section with strain added is labelled as S2, while the first 921 m loose fibre is labelled as S1.

For one vibration result, the BFS profiles are measured 124 times and the vibration information for a specific location is extracted from the 124 BFS profiles. Each measurement lasts for 50 ms. After STFT of the captured time domain sensing data is performed, BGSs along the FUT are obtained. These BGSs along the FUT are illustrated in images. With the horizontal axis and the vertical axis representing the fibre length and the BGS frequency, respectively, the colour of the images indicates the amplitude of each point on the BGSs. Noise removal is conducted on the 124 derived BGS images with the trained CNN for one vibration detection. The BFS profiles are then extracted from the denoised images by finding the centre frequencies of the BGSs. The strain vibration profiles of the optic fibre are then detected from the new denoised BFS. 

### 2.3. Training Setup

The Berkeley segmentation dataset (BSDS300) of size 200 by 200 is used to train the network as clean images [22]. Gaussian noise with standard deviation of 110 is added to the images of the dataset to generate noisy input images. Since the training target is the noise but not the clean image, the BSDS300 is chosen instead of simulated BGS images. The BSDS300 dataset includes more variations of details than the simulated BGS images, and hence the network can be better trained. Simulated BGS images were tested as the training set, but the outcomes were even worse than the original BFS results. This is due to the fact that real BGS images include more complicated BFS variations than simulated BGS images. On the other hand, if there was enough experimental data, the experimental dataset could be used as the training dataset. 

The filter weights are initialized by the method in [28] and the batch size of 4 is used. The depth and the layers are described as follows. The first layer—1 convolutional layer with ReLU: 64 filters of size 3 by 3 are used to generate 64 feature maps. The second type of layers with depth D–D convolutional layers with BN and ReLU: 64 filters of size 3 by 3 by 64 are used for each layer. The last layer—1 convolutional layer: a filter of size 3 by 3 by 64 is used to reconstruct the output. The depth D is to be investigated in the experiments. The total depth of the network is (D + 2). The choices of the depths and the epoch numbers are experimentally studied in Section 3.

## 3. Results and Discussion

### 3.1. Experiments with Different Total Depths and Epochs

To compare the performance of the neural network with different depths and epochs, the total depth of the hidden layers is set to 4, 8, 12, 16, respectively, while the epoch is set to 50 and 200, respectively. The depth of the second type hidden layers is therefore 2, 6, 10, 14, respectively. The neural network is trained eight times in total with the set depths and epochs. The 124 BGS images are denoised using each trained network.

**Figure 4:**
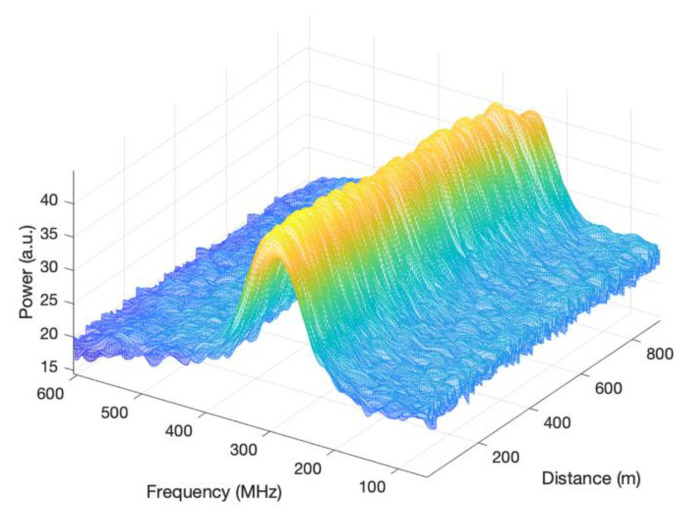
Three-dimensional map of the measured BGSs.

Figure 4 shows a three-dimensional map of the measured BGSs along the fibre distance. The top view of the map is the BGS image to be denoised.

The training loss curves with different total depths and epoch numbers are shown in Figure A2, Appendix B. Good convergence can be seen in all figures in Figure A2, indicating reasonable training of the models. 

**Figure A1:**
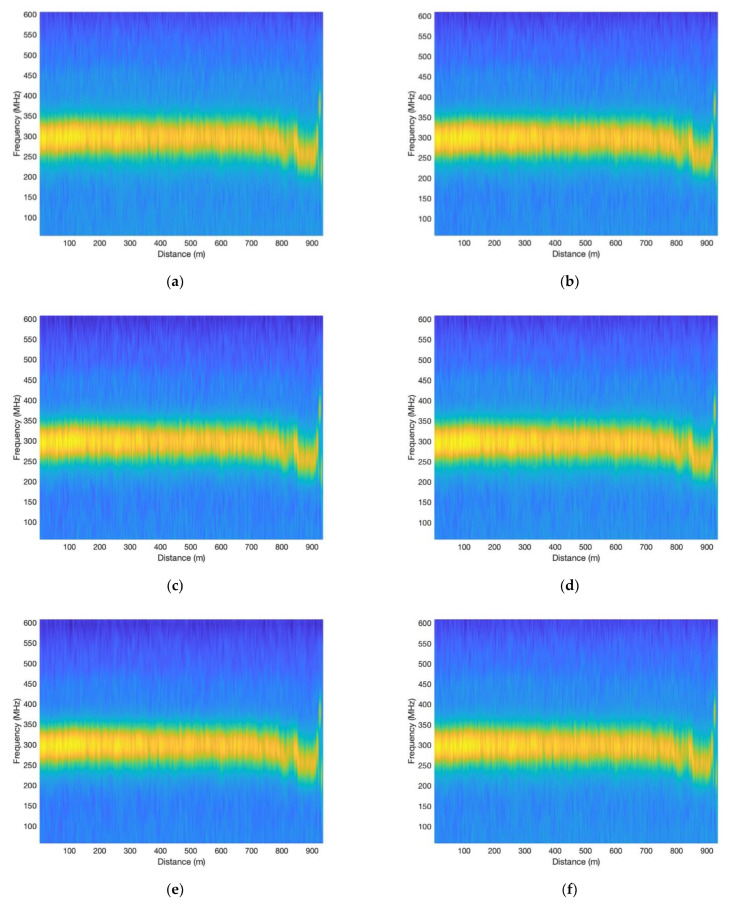

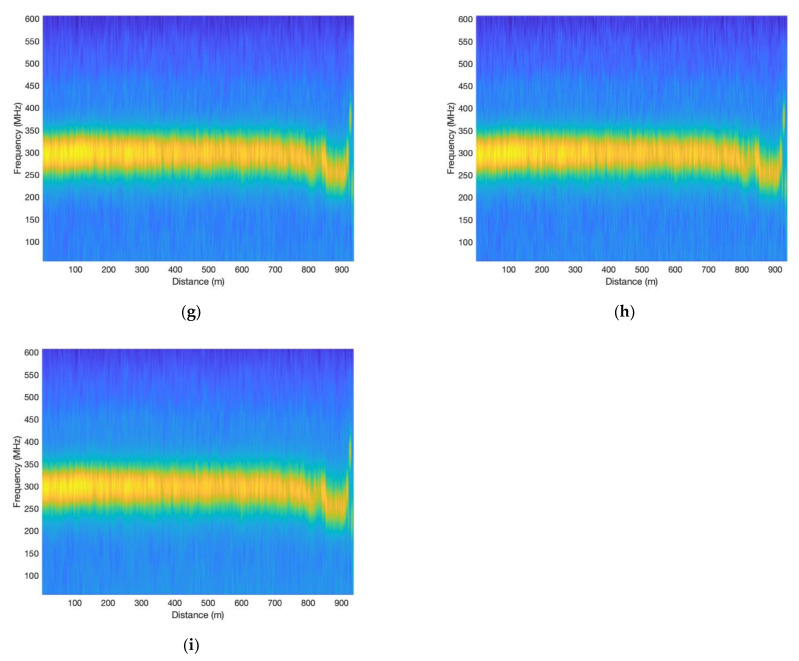
The BGS image without denoising, compared with the denoised BGS images with different total depths and epochs. (**a**) BGS spectra without denoising. (**b**) Denoised BGS spectra with total depth = 4, epoch = 50. (**c**) Denoised BGS spectra with total depth = 4, epoch = 200. (**d**) Denoised BGS spectra with total depth = 8, epoch = 50. (**e**) Denoised BGS spectra with total depth = 8, epoch = 200. (**f**) Denoised BGS spectra with total depth = 12, epoch = 50. (**g**) Denoised BGS spectra with total depth = 12, epoch = 200. (**h**) Denoised BGS spectra with total depth = 16, epoch = 50. (**i**) Denoised BGS spectra with total depth = 16, epoch = 200.

**Figure A2:**
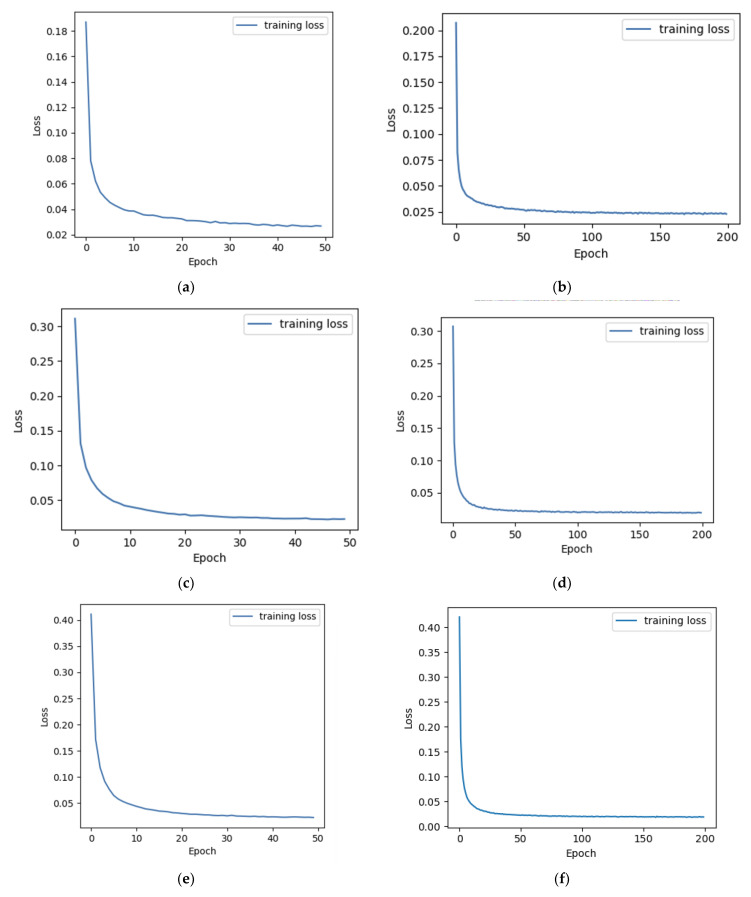

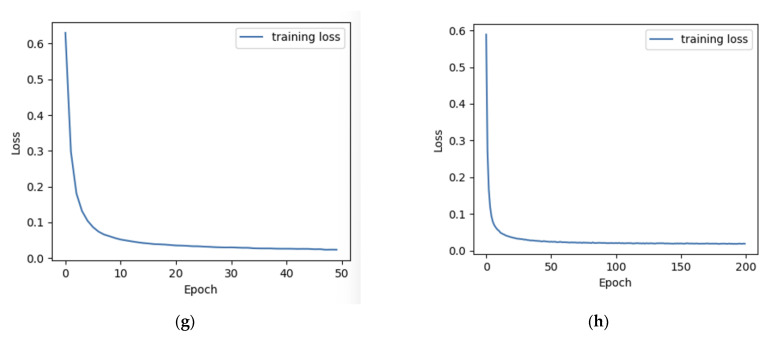
The training loss curves with different depths and epoch numbers. (**a**) Loss curve with total depth = 4, epoch = 50. (**b**) Loss curve with total depth = 4, epoch = 200. (**c**) Loss curve with total depth = 8, epoch = 50. (**d**) Loss curve with total depth = 8, epoch = 200. (**e**) Loss curve with total depth = 12, epoch = 50. (**f**) Loss curve with total depth = 12, epoch = 200. (**g**) Loss curve with total depth = 16, epoch = 50. (**h**) Loss curve with total depth = 16, epoch = 200.

The BGS image without DnCNN is shown in Figure A1a in Appendix A. The denoised BGS images along the FUT, with different total depths and epochs of DnCNN are demonstrated in Figure A1b–i. It should be noted that there are 124 denoised images for each set total depth and epoch. Figure A1 illustrates one single image from the 124 images for each training network. The centre yellow parts of the figures are the BGSs. Upshifts of BGS frequencies are observed at the far end of the FUT, which are caused by the applied strain onto the fibre. Clearer images are seen with an epoch of 200 than those with an epoch of 50, when the total depth of the network is fixed. Figure A1a is the fuzziest without DnCNN denoising. 

**Figure 5:**
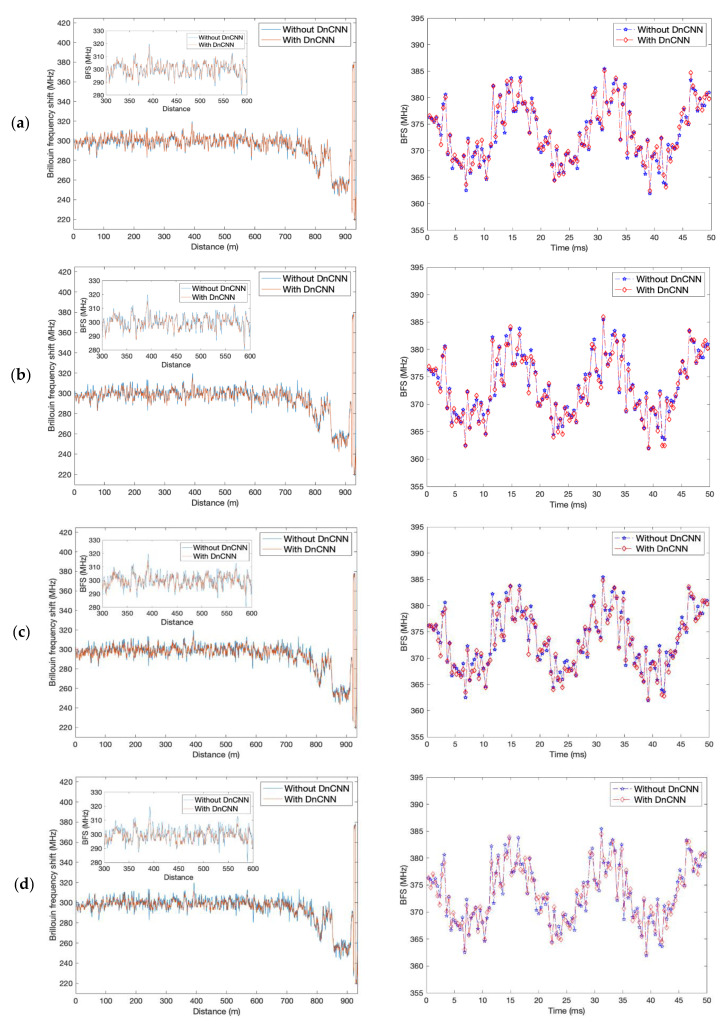
Left: BFS along the FUT obtained from the denoised BGS spectra (red), compared with the BFS from the BGS spectra without denoising (blue). Right: strain vibration profile with DnCNN denoising (red), compared with the strain vibration profile without denoising (blue). (**a**) Depth = 4, epoch = 50; (**b**) depth = 4, epoch = 200; (**c**) depth = 8, epoch = 50; (**d**) depth = 8, epoch = 200.

**Figure 6:**
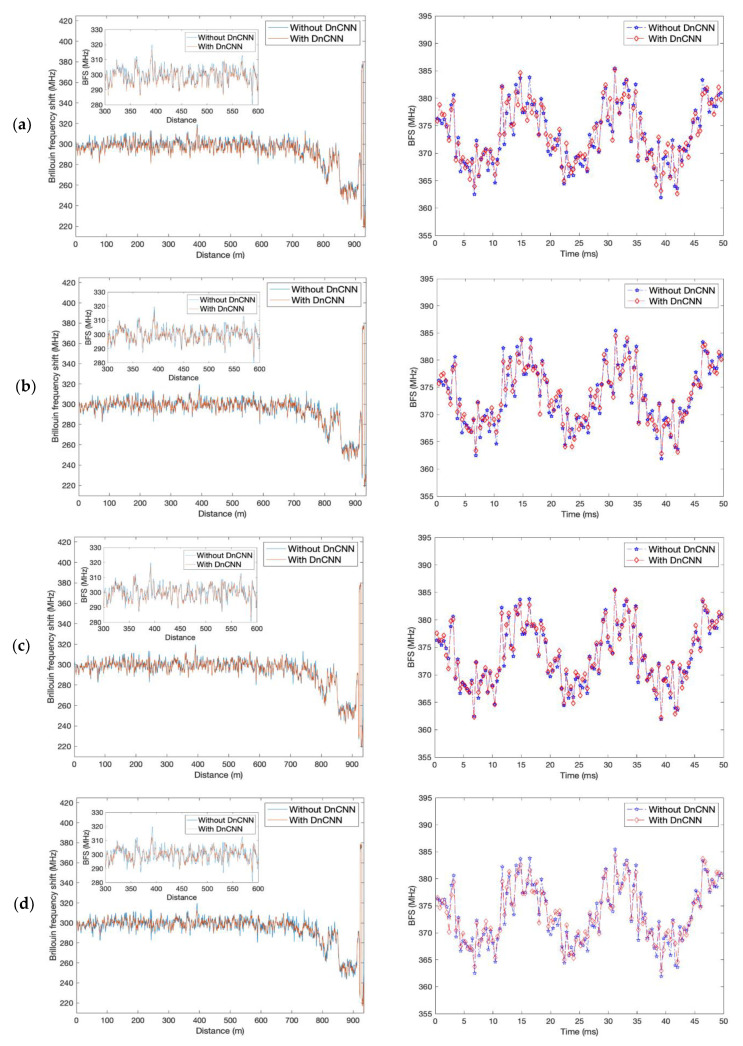
Left: BFS along the FUT obtained from the denoised BGS spectra (red), compared with the BFS from the BGS spectra without denoising (blue). Right: strain vibration profile with DnCNN denoising (red), compared with the strain vibration profile without denoising (blue). (**a**) Depth = 12, epoch = 50; (**b**) depth = 12, epoch = 200; (**c**) depth = 16, epoch = 50; (**d**) depth = 16, epoch = 200.

The detected BFS profiles along the FUT for one of the 124 measurements are shown on the left in Figure 5 and Figure 6 with different total depths and epochs of the DnCNN network (red), and the BFS profile without denoising (blue) is illustrated in each figure as a comparison. The insets of the left figures in Figure 5 and Figure 6 are the zoomed profiles from 300 m to 600 m of each figure. The strain changes can be clearly seen at the far end of the FUT in each figure. All the figures show smoother BFS profiles with DnCNN denoising compared with the profiles without denoising, with frequency uncertainty improvements between 0.17 MHz to 1.48 MHz. With the same total depth, the epoch number of 200 demonstrates less fluctuant BFS profiles compared with the epoch number of 50. Total depths of 8 and 16 lead to relatively smoother BFS profiles, leading to 1.22 MHz and 1.48 MHz frequency uncertainty improvements, respectively, with 200 epochs. Whereas BFS profiles with a total depth of 4 induce only 0.17 MHz frequency uncertainty improvement with 50 epochs and 0.68 MHz improvement with 200 epochs, which are the worst results for both epoch number settings.

By examining the 124 BFS profiles, the 60 Hz strain vibration over 50 ms of time is detected. The vibration profiles with DnCNN denoising at different total depths and epochs (red) are drawn in the right figures in Figure 5 and Figure 6, compared with the detected strain vibration without denoising (blue). The peak-to-peak change in BFS is about 16 MHz, corresponding to 320 με strain change on the fibre. The improvement of the vibration detection can be slightly more obvious in Figure 5d and Figure 6b,d by visual inspection but is not intuitionistic enough.

**Figure 7:**
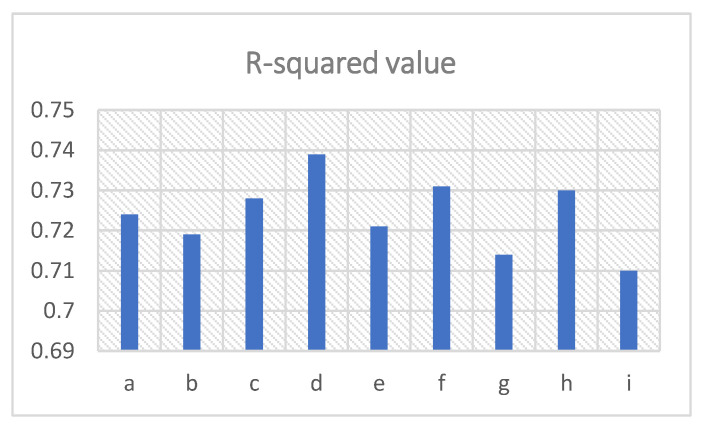
R-squared values of sine fitting for the detected strain vibrations with DnCNN denoising at different total depths and epochs (column a to h), compared with the strain vibration without denoising (column i). (The numbers a to i match the first column in Table 1).

**Figure 8:**
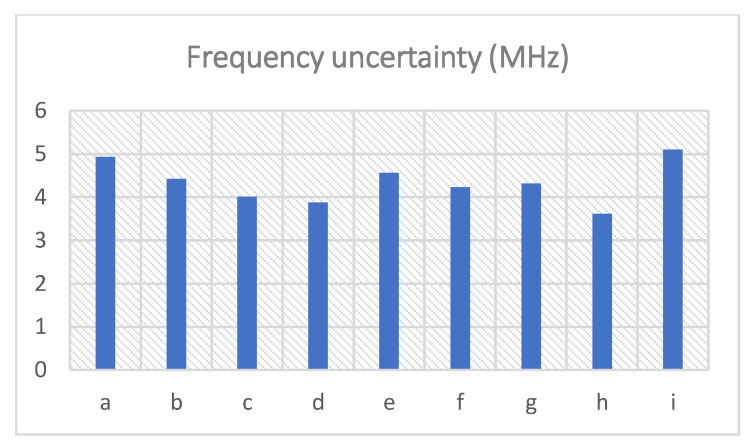
Frequency uncertainties of BFS with DnCNN denoising at different total depths and epochs (column a to h), compared with the frequency uncertainty without denoising (column i). (The numbers a to i match the first column in Table 1).

To verify the performance of the noise reduction on the strain vibration detection, the measured vibration results are sine fitted and the R-squared values of the sine fitting are derived for each given total depth and epoch. The R-squared value is a statistical measure that shows how well the dataset fits the regression model. It determines the proportion of the variance in the dependent variable that can be explained by the independent variable. Its value ranges from 0 to 1, and the larger the value, the better the correlation between the fitting model and the data. The frequency uncertainty of the BFS is calculated as the standard deviation of the BFS over time. The R-squared values and the frequency uncertainties are written in Table 1 for each total depth and epoch number of the DnCNN. The R-squared value and the frequency uncertainty of the measured result without denoising are also listed in Table 1 (No. i) as a comparison. The results with different depths and epochs are numbered in Table 1 (as a to h). The corresponding R-squared values and the frequency uncertainties are shown in the charts in Figure 7 and Figure 8, respectively. The column numbers of a to i agree with Table 1.

It can be observed in Table 1 and Figure 7 that the DnCNN with a total depth of 8 and epoch of 200 provides the best performance for the detection of sinusoidal strain vibration which has the largest R-squared value among all the results. With the total depth of 8, 12 and 16, the R-squared values with 200 epochs are higher than the results with 50 epochs. With a total depth of 4, the R-squared value of 50 epochs is slightly larger than that of 200 epochs. On the other hand, comparing the results with the same epoch number, the R-squared values with a total depth of eight are the most enhanced. The R-squared values with a total depth of 12 and 16 are almost the same as the second best, whereas the R-squared values with a total depth of 4 are the lowest. All the R-squared values of sine fitting for the detected strain vibrations from denoised BGS images are better than the value without denoising.

From Table 1 and Figure 8, the frequency uncertainties with the total depth of 8 and 16 and the epoch number of 200 are the most decreased among all the results, which are reduced to below 4 MHz. With the same total depths (4, 8, 12 or 16), the frequency uncertainties with 200 epochs are lower and hence better than those with 50 epochs. Moreover, the frequency uncertainty with a total depth of 16 is the smallest for 200 epochs, and the frequency uncertainty with a total depth of 8 is the smallest for 50 epochs. On the contrary, the frequency uncertainty with the depth of 4 and 12 and the two largest, whichever the epoch number is. Furthermore, all the obtained frequency uncertainties with DnCNN denoising are improved compared with that with no denoising. 

Comparing the performance with the same total depth, generally, all the experimental results with 200 epochs are better than those with 50 epochs, although there is some disturbance for the depth of 4. With a batch size of 4 and a training dataset of 200, the trainable parameters are updated 50 times for each epoch. With a larger number of epochs, the parameters (the weights) are updated more times and hence better convergence is realised. The loss function is better minimized and the denoising performance is also better, at the expense of time consumption. On the other hand, as the epoch number goes further larger, the network training loss tends to be flattened and the improvement of the denoising performance is limited. 200 epochs can already provide noticeable training performance [22], and a larger epoch number will take up more processing time without significant performance improvement. 

On the other hand, by comparing the experimental results with the same epoch number, it can be seen that as the total depth increases from 4 to 16, the detected results get first better and then worse and then better again. In a deep learning network, each layer progressively extracts higher level features of the input until the final layer, from the edges, object parts to objects [29]. With four hidden layers, the DnCNN extracts relatively lower level features, and the network is not yet trained to obtain enough features of the target BGS images. With the network going deeper to eight layers, more complex features of the input are extracted. The network performs better training and better denoising of the images. As the total depth goes even deeper to 12 and 16, there are even more complex features extracted. However, there are also more trainable parameters introduced into the network. Hence, the training process of the network is more complicated as well. Different from normal natural images, the BGS images are made up of a large number of spectra that have similar features and shapes. Additionally, their features are not as complicated as the natural image. With a total depth of 8, the features can already be extracted well. With a deeper network, the BGS images are overfitted. From Figure 6, the denoised BFS profile in Figure 6 (d) is smoother, but some details are suppressed. Since the network with a depth of 8 and 200 epochs can generate comparable frequency uncertainty while keeping more details of the BFS, the DnCNN with 8 layers and 200 epochs is the most feasible for the denoising of BGS images along the FUT. 

### 3.2. Spatial Performance and the Brillouin Gain Spectra

**Figure 9:**
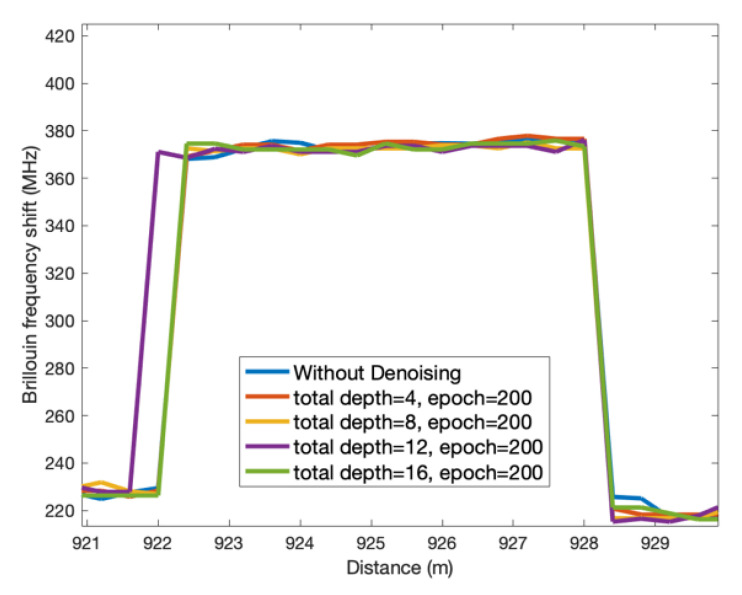
The BFS profiles of the optic fibre section S2.

To investigate the spatial performance with DnCNN denoising, Figure 9 illustrates measured BFS profiles of the optic fibre section S2 with strain applied at different total depths and 200 epochs. The change in BFS due to the applied strain can be observed from all the profiles from 922 m to 928.4 m, except the profile with 12 layers. The rising point of the detected strain is one point forward with 12 layers. Due to the double peak effect where there is a strain change, distance error might be induced after denoising as the Brillouin amplitudes of the two peaks can be comparable and the denoising is based on the whole image with spatial context information considered [30]. 

**Figure 10:**
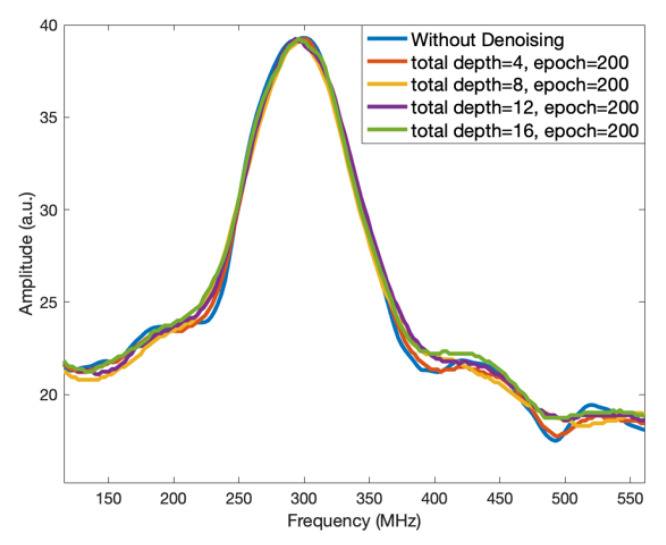
The DnCNN denoised BGS spectra.

The obtained BGSs with DnCNN denoising and without denoising are shown in Figure 10, respectively. It can be observed that the fluctuations in the spectra are reduced after denoising compared with the spectrum without denoising although the noise level is not significantly suppressed. In addition, there are shifts in the peak BGS frequencies with different depths after denoising. In the Brillouin-based distributed fibre optic sensors, there is another common definition of SNR which is better related to the performance of a system as the ratio of the mean BFS signal to the standard deviation of the BFS signal [7]. Hence, the suppression of the fluctuation of BGSs improves the SNR and can lead to more accurate detection of BFS. 

### 3.3. Comparison with Some Known Methods

Table 2 demonstrates the experimental results of Brillouin-based fibre optic sensors with some published image denoising methods, compared with the results of this work. It can be seen that the image denoising methods used are mainly NLM, WD and BM3D. The BFS uncertainty can be improved within the range of 57.2% to 23.9% of the original value with these three methods based on the different denoising optimization parameters chosen as well as different fibre lengths, input pulse widths (the nominal spatial resolution) and different averaging numbers of the traces. Nevertheless, the first three lines of Table 2 show the degradation of these denoising methods, which can be very significant to as large as 23 times the nominal frequency uncertainty, due to the missing of data details as the SNR of signal increases [1]. The spatial resolution can also broaden to over twice the original value. In addition, these methods require complex parameter adjustment [3,8]. In addition, the performance of NLM depends on the similarity of the neighbouring data. Moreover, due to the confusion of the definition of frequency uncertainty, many are using the distance domain approach, i.e., the standard deviation of the BFS along the fibre from one measurement within a certain spatial window length, which is inaccurate and overestimates the BFS uncertainty, making the results much better than the real values [6]. In fact, the sequential domain approach, that is, calculating the standard deviation at each fibre location over consecutive measurements is the more accurate method [6]. However, in many publications the calculation approaches of the uncertainty are not clearly defined, making it difficult to compare the performance of different works. In addition, for many static measurements, only a single experimental result is obtained after averaging, so it can be inferred that the distance domain approach is used since there are not enough data for the sequential domain approach. In this work, the sequential domain approach is applied. All the referred results in Table 2 are based on static measurement, and it is suspected that the distance domain approach is used for most of these measurements. Since the distance domain approach can lead to about 2.6 times the overestimation of frequency uncertainty, which is discussed in [6], it is hard to compare the real performance of Table 2. 

On the other hand, compared with BOTDA, the Brillouin signal power from BOTDR is exponentially smaller, making the signal much harder to be detected and the SNR worse. Furthermore, conventional Brillouin-based fibre optic sensors use the frequency sweeping method to acquire the data, which is time-consuming and limits the performance with the frequency step. STFT is a much faster way to obtain the frequency domain data while keeping the redundant information and conducting a denoising effect on the data. The second line from the bottom in Table 2 is the result with STFT and WD [31]. The frequency uncertainty is improved by 19% for this experiment. However, the uncertainty of our work is improved by 24%, which is better than [31], indicating more effective denoising performance. Although the absolute value of [31] is smaller, the spatial resolution of [31] is 5 times the value of this work, and the averaging number is 16 of our work. Considering the enhancement of SNR with these larger values and the possible distance domain approach for frequency uncertainty, the smaller absolute values cannot stand for necessarily better results. 

Still, all other experiments in Table 2 are static measurements, apart from our work. For our work, the dynamic measurement sampling rate is 2.5 kHz, indicating the capability of vibration detection up to 1.25 kHz. In addition, DnCNN denoising is applied based on the results with STFT processing, polynomial fitting and the induced small gain SBS, which have already performed some denoising effects. The DnCNN here further improves the system performance and helps to enhance the vibration detection results.

The availability of experimental data could be a limitation of this study. If there are enough experimental data, real noisy data could be used as the training dataset in a later study, so that the training set could be more related to the validation dataset. In addition, experiments with different vibration speeds and amplitudes could better verify the denoising model. In addition, as deep learning is under rapid development, better results might be realized with other technologies.

## 4. Conclusions

In this work, the image denoising method of DnCNN is introduced and trained to reduce the noise of the BGS images and improve the performance of vibration detection of the small gain SBS STFT-BOTDR. To investigate the influence of the network depth and the epoch number on the denoising performance of the BOTDR system, the total depth is set to 4, 8, 12 and 16, respectively, and the epoch number is set to 50 and 200, respectively. 

By denoising of the BGS images along the fibre length, experimental results show that the total depths of 8 and 16 with the epoch number of 200 generate the best denoised outputs for the BOTDR system in terms of the BFS frequency uncertainties (improved by 1.22 MHz and 1.48 MHz, respectively), and the sine fitting R-squared values (improved by 0.029 and 0.02, respectively), of the detected vibration when denoising is conducted on the BGS images. The obtained BFS profiles along the FUT are also less fluctuant in these cases. However, to better maintain the details and avoid over denoising, the total depth of 8 and epoch number 200 are the best choices, with the frequency uncertainty improved to 3.88 MHz.

As the averaging number is a significant determinant of the dynamic sensing speed, the averaging number must be controlled to a small value to realise the dynamic measurement of BOTDR [27]. On the other hand, a small averaging number leads to more noise, especially for a BOTDR system, where the Brillouin signal is weak, and the SNR is very limited. Therefore, noise reduction is important for the dynamic BOTDR system to enhance the frequency uncertainty. In this work, the averaging number is set to 25, leading to a 2.5 kHz dynamic measurement sampling rate for a 16 µs pulse period. By involving DnCNN, the frequency uncertainty of small gain SBS STFT-BOTDR measurement can be improved by 24%, and the vibration detection can be improved to 0.739 from 0.710 in terms of the R-squared fitting value. In addition, the conventional image denoising methods, such as WD, NLM and BM3D, can result in unneglectable degradation, due to the loss of information. The frequency degradation can be as large as over 10 MHz, and the spatial resolution can be broadened to as wide as over twice of the original value. DnCNN keeps the redundancy of the information and overcomes this drawback. Hence, with DnCNN, the system can produce more accurate BFS information and therefore more accurate vibration detection, which can be applied in fields such as earthquake detection and intrusion detection.

## Figures and Tables

**Table 1:** The R-squared values of sine fitting and the frequency uncertainties of BFS for different total depths and epochs of DnCNN networks.

No.	Depth	Epoch	R-Squared	Frequency Uncertainty (MHz)
a	4	50	0.724	4.93
b	4	200	0.719	4.42
c	8	50	0.728	4.01
d	8	200	0.739	3.88
e	12	50	0.721	4.56
f	12	200	0.731	4.23
g	16	50	0.714	4.31
h	16	200	0.730	3.62
i	-	-	0.710	5.10

**Table 2:** Performance comparison with some other known methods.

Method	Setup	BFS Uncertainty/Accuracy	Original BFS Uncertainty	Spatial Resolution	Fibre Length	Averaging Number	BGS Acquisition	Fast Measurement Sampling Rate	Fibre Vibration Speed
NLM [1]	BOTDA	0.57 °C/13.32 °C ^1^	-	2 m/4.42 m ^2^	62.3 km	16	Frequency scanning	-	-
WD [1]	BOTDA	0.55 °C/8.81 °C ^1^	-	2 m/5.5 m ^2^	62.3 km	16	Frequency scanning	-	-
BM3D [1]	BOTDA	0.55 °C/2.17 °C ^1^	-	2 m/3.86 m ^2^	62.3 km	16	Frequency scanning	-	-
NLM [2]	BOTDA	0.843 MHz	1.473 MHz	4 m	40.63 km	1	Frequency scanning	-	-
NLM [3]	BOTDA	0.77 MHz	-	2 m	100 km	2000	Frequency scanning	-	-
NLM [5,6]	BOTDA	1.2 MHz	4.5 MHz	2 m	50 km	4	Frequency scanning	-	-
WD [5,6]	BOTDA	1.3 MHz	4.5 MHz	2 m	50 km	4	Frequency scanning	-	-
BM3D [8]	BOTDA	2.1 °C	8.8 °C	2.5 m	100.8 km	2000	Frequency scanning	-	-
STFT and WD [31]	BOTDR	1.27 MHz	1.57 MHz	20 m	12.5 km	400	STFT	-	-
This work	BOTDR	3.88 MHz	5.1 MHz	4 m	935 m	25	STFT	2.5 kHz	60 Hz

^1^ The first value is the nominal frequency uncertainty, while the second value is the accuracy degradation. The temperature values are proportional to BFS uncertainty for Brillouin-based sensors, and 1 °C approximates 1 MHz. ^2^ The first value is the nominal spatial resolution, while the second value is the degraded spatial resolution.

## Data Availability

The data presented in this study are available on request from the corresponding author. The data are not publicly available due to potential commercial values.
